# Efficacy of Dexamethasone and Tranexamic Acid in Reducing Bleeding, Edema, and Ecchymosis in Rhinoplasty Patients: A Systematic Review and Meta-Analysis

**DOI:** 10.7759/cureus.96698

**Published:** 2025-11-12

**Authors:** Hosam Hadi Hassan Awaji, Saud A AlShnabir, Omar S Alzamil, Atallah A Almadi, Mohammed S Al ghamdi, Fares A Alhejaili, Osama Almohammadi, Nouf Alsharef, Faisal A Alshyer, Ahmed H Alkhaldi, Amal K Almutairi, Aljoharh A Alnuaman

**Affiliations:** 1 Preventive Medicine, North West Armed Forces Hospitals, Tabuk, SAU; 2 Faculty of Medicine, Imam Abdulrahman Bin Faisal University, Dammam, SAU; 3 Otolaryngology - Head and Neck Surgery, University of Tabuk, Tabuk, SAU; 4 Faculty of Medicine, Batterjee Medical College, Abha, SAU; 5 Faculty of Medicine, Ibn Sina National College, Jeddah, SAU; 6 Faculty of Medicine, Batterjee Medical College, Jeddah, SAU; 7 Emergency Department, Khamis Mushait General Hospital, Khamis Mushait, SAU; 8 Faculty of Medicine, Al Jouf University, Al Jouf, SAU; 9 Faculty of Medicine, Majmaah University, Al Majmaah, SAU; 10 Emergency Department, King Abdulaziz Specialist Hospital, Taif, SAU

**Keywords:** bleeding, dexamethasone, ecchymosis, edema, meta-analyses, rcts, rhinoplasty, tranexamic acid

## Abstract

This study aims to evaluate the efficacy of dexamethasone and tranexamic acid, or a combination of both, in reducing intraoperative bleeding, postoperative edema, and ecchymosis in patients undergoing rhinoplasty. The present review searched PubMed/Medline, ClinicalTrials.gov, and Scopus. The study included randomized clinical trials (RCTs). The efficacy assessment was conducted in terms of intraoperative blood loss, edema score, and ecchymosis score. To evaluate the risk of bias (ROB), the revised version of the Cochrane ROB tool was used. Nine eligible RCTs, including 484 patients undergoing rhinoplasty, were assessed. The overall random-effects pooled prevalence of intraoperative blood reduction was -22.01 ml (95% CI: -34.93 to -9.08; P <0.001). The edema score was -0.87 (95% CI: -1.07 to -0.67, P <0.001), and the ecchymosis score was -0.99 (95% CI: -1.37 to -0.61; P <0.001). The overall quality of the ROB can be moderate, as most studies demonstrated a low ROB in critical domains, but some exhibited concerns in areas such as allocation concealment and blinding, with one study showing a high ROB. The findings indicated that tranexamic acid and dexamethasone significantly improved patient outcomes because they decreased intraoperative bleeding, edema, and ecchymosis. These findings highly suggest that tranexamic acid and dexamethasone should be administered during rhinoplasty.

## Introduction and background

Rhinoplasty is a surgical procedure utilized to alter and reconstruct nasal structures for cosmetic and functional purposes [1]. The common side effects of rhinoplasty are intraoperative blood loss, postoperative edema, and ecchymosis, which can lead to psychological and physical stress on patients [2]. Blood loss may lead to a subsequent need for blood transfusion, and the blood used for transfusions has a limited shelf life and a high processing cost. Moreover, blood transfusion is possibly associated with transmitting blood-borne diseases, infections, and other complications [3].

The balance between coagulation and fibrinolysis must be maintained to prevent bleeding and maintain circulation during surgery, and a hypotensive anesthesia approach [4] has been used to reduce intra- and postoperative blood loss. Despite some reports suggesting that hypotensive anesthesia may raise the risk of cerebrovascular accidents, the procedure has been considered safe. A new strategy to minimize rhinoplastic blood loss is to use antifibrinolytic medications, such as tranexamic acid (TXA), aprotinin, and aminocaproic acid, preoperatively. TXA is used to stabilize microclots formed at a surgical wound. These antifibrinolytic agents can help create a clean surgical field to have good intraoperative visualization [5]. TXA is a safe antifibrinolytic agent and is considered a lysine analog. It prevents the conversion of plasminogen into plasmin and has been confirmed to minimize bleeding during surgery and the need for blood transfusion [6,7].

The anti-inflammatory characteristics of the corticosteroids result in a decrease in vascular permeability, giving rise to diminution of exudates and edema. Some studies have already reported the favorable effects of corticosteroid usage for this purpose [3,8]. Dexamethasone administration might decrease the onset of ecchymosis and edema due to its great anti-inflammatory properties, and it has the potential to bind with chromatin located in the cellular nucleus and influence gene expression to synthesize various anti-inflammatory enzymatic proteins. By stabilizing the cell membrane and inhibiting fibroplasia, these proteins contribute to the reduction of inflammation. Furthermore, dexamethasone's ability to reduce vascular permeability has also been reported to be an additional important reason, owing to which it delays the onset of edema and ecchymosis effectively [9].

The aim of this systematic review and meta-analysis is to evaluate the effectiveness and the role of TXA and dexamethasone alone or in combination with each other in reducing intraoperative blood loss, postoperative edema, and ecchymosis. Also, based on high-quality evidence, it aimed to update readers on the most recent findings and clinical guidelines related to the effectiveness of TXA and dexamethasone in rhinoplasty.

## Review

Methodology

The study followed the guidelines outlined in the Cochrane Handbook for Systematic Reviews of Interventions, version 6. The results were reported following the Preferred Reporting Items for Systematic Reviews and Meta-Analyses (PRISMA) guidelines [10].

Study Question

What is the efficacy of dexamethasone and tranexamic acid in reducing intraoperative blood loss, postoperative edema, and ecchymosis in patients undergoing rhinoplasty?

Search Strategies and Selection Criteria

The articles were searched on several databases for the present systematic review, including PubMed, Medline, Scopus, and ClinicalTrials.gov. The following keywords were used: (“Rhinoplasty” OR “Nasal Surgery’ ’AND “Dexamethasone” OR “Corticosteroids” AND ‘’Tranexamic Acid” OR “Antifibrinolytic Agents “AND “Bleeding” OR “Hemorrhage” AND ‘’Edema” OR “Swelling” AND “Ecchymosis” OR “Bruising”). Two authors independently evaluated the search and review process to ensure the accuracy and reliability of the findings.

Eligibility Criteria

Inclusion criteria: The inclusion criteria were (1) rhinoplasty patients who received tranexamic acid and/or dexamethasone as an intervention; (2) at least one of the outcomes was reported: total loss of blood, hemoglobin levels, drainage output, edema score, ecchymosis score, and hematoma; (3) studies conducted in the last 10 years; (4) RCTs (study design); (5) studies in the English language.

Exclusion criteria: The exclusion criteria were (1) studies that had qualitative data only; (2) letters, reviews, editorials, observational, retrospective studies, and abstracts; (3) studies that did not assess the main outcomes.

Study Selection

Two separate researchers carried out the procedures of searching the internet, reviewing the complete texts of pertinent papers, and filtering titles and abstracts. Consensus was used to resolve any disagreements.

Data Extraction

The data extraction process was performed by two authors via Microsoft Excel (Microsoft Corporation, Redmond, WA) [11]. Name of the author and publication year, location, study design, age, interventional treatment, control treatment, primary outcomes, and the study results were extracted from the studies. Variables were calculated from the original data when the information was not clearly stated.

Quality Assessment of the Included Studies

We assessed the risk of bias (ROB) in the selected studies using the revised version of the Cochrane Risk of Bias 2 (ROB2) tool for RCTs [12]. The ROB2 tool comprises five domains: randomization, deviations from the assigned treatment, missing outcome data, measurement of the outcome, and selective reporting of the outcomes and results. Furthermore, the overall ROB was evaluated by selecting the highest level of ROB from the five domains. The figures were visualized using the Robvis tool [13]. Studies were evaluated according to the ROB tool as having a “low risk,” “high risk,” or “some concerns.”

Outcomes Measures

The mean scores of ecchymosis, edema, and mean intraoperative blood loss were distributed and analyzed separately.

Statistical Analysis

The meta-analysis was conducted using RevMan (Cochrane Collaboration, London, UK) to generate forest plots of the effect estimates of the included studies [14], and 95% confidence intervals were calculated. The extracted data were described using the mean and standard deviation (SD). The meta-analysis converter tool was used to approximate the mean and SD when the median and range or interquartile range (IQR) were inclined [15]. The overall pooled random-effect model was used to synthesize data for relevant outcomes. The P-value was considered statistically significant if it was lower than 0.05. The heterogeneity was evaluated using I^2^ statistics. If I^2^ was ≤50%, an investigation of the potential source of heterogeneity related to both methodological and clinical aspects of the studies was conducted. The systematic review included a specific number of studies that were screened and selected.

Results

**Figure 1:**
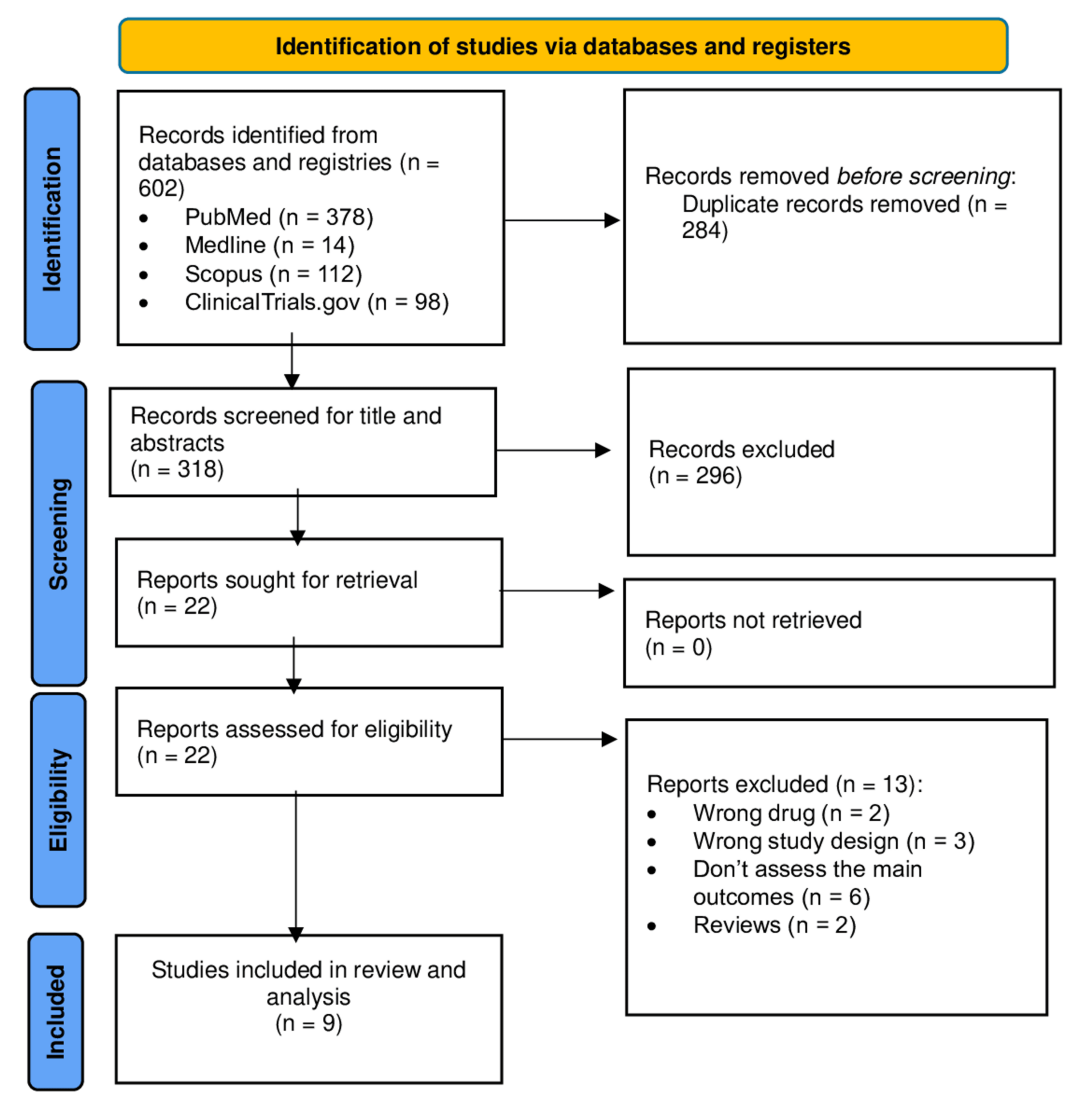
PRISMA flowchart of the number of studies screened and included in the systematic review. PRISMA: Preferred Reporting Items for Systematic Reviews and Meta-Analyses.

The screening and selection process yielded a total of 602 articles. After filtering the data, 318 records were screened based on title/abstract, and 22 articles were retrieved as possible articles for full-text screening. Thirteen papers were excluded due to the following reasons: wrong drug (n = 2), wrong study design (n = 3), did not assess the main outcomes (n = 6), and review articles (n = 2). Considering the predefined inclusion and exclusion criteria, a total of nine studies were included in this systematic review. The PRISMA flowchart (Figure 1) shows the detailed steps of the screening process [3,5,16-22].

Characteristics of the Included Studies

All studies included in the review were randomized clinical trials (RCTs), having a total of 484 patients. The intervention groups were treated with tranexamic acid and/or dexamethasone. The average age of the participants ranged between 16 and 49 years. Five studies were conducted in Iran, two in Turkey, one in Bahrain, and one in Brazil. The main outcomes (intraoperative blood loss, postoperative edema, and ecchymosis severity scores) were present among the included trials, except in two studies [3,22], which included intraoperative bleeding only. Table 1 details the main characteristics of the included studies.

**Table 1:** Characteristics of the included studies. TXA: tranexamic acid; RCT: randomized controlled trial.

Author	Year	County	Study design	Sample size	Age, mean (range), years	Intervention group (TXA, dexamethasone, or both)	Control group	Outcomes	Results
Beikaei et al. (2015) [22]	2015	Iran	Prospective randomized double-blinded RCT	96 (48 TXA, 48 controls)	16-42	10 mg/kg TXA	Placebo	Intraoperative blood loss	Decreases intra-operative bleeding with a 15.6 mL decrease (95% CI: 11.8-19.4, p < 0.001) in intraoperative bleeding
Eftekharian et al. (2016) [3]	2016	Iran	RCT	50 (25 TXA, 25 controls)	16-40	1.0 g/kg TXA	Placebo	Intraoperative blood loss	The preoperative administration of 1 g oral TXA significantly decreased the blood loss in patients undergoing rhinoplastic surgery without any significant adverse effects
Ghavimi et al. (2017) [21]	2017	Iran	Double-blind RCT	50 (24 TXA, 26 controls)	19-40	10 mg/kg TXA	Placebo	Intraoperative blood loss, postoperative edema, and ecchymosis severity scores	Administering 10 mm/kg of TXA significantly reduced intraoperative bleeding, eyelid swelling, and periorbital bruising during rhinoplasty with minimal side effects
Valente et al. (2015) [17]	2015	Brazil	A randomized, double-blind, placebo-controlled clinical trial	42 (20 dexamethasone, 22 controls)	23.1 ± 5	10 mg/kg dexamethasone	Saline	Postoperative edema and ecchymosis severity scores	Significant decrease in ecchymosis and edema in the experimental group as compared to controls.
Mehdizadeh et al. (2018) [16]	2018	Iran	Prospective randomized triple-blinded RCT	60 patients ( 15 TXA, 15 dexamethasone, 15 TXA and dexamethasone, and 15 controls)	18-39	TXA = 10 mg/kg, dexamethasone = 8 mg/kg, TD = 10.8 mg/kg i/v	Placebo	Postoperative edema and ecchymosis severity scores	TXA and dexamethasone, separately or in combination, had similar effects in reducing periorbital edema and ecchymosis in open rhinoplasty compared to controls
Sakallioğlu et al. (2015) [5]	2015	Turkey	RCT	50 (25 TXA, 25 control)	20-36	1 g/kg TXA	Placebo	Intra-operative blood loss, postoperative edema, and ecchymosis severity scores	Marked decline in blood loss, edema, and ecchymosis in TXA as compared to controls
Haddady-Abianeh et al. (2022) [19]	2022	Iran	Prospective randomized double-blinded clinical trial	28 (14 TXA, 14 control)	18-49 (30.67 ± 8.27)	10 mg/kg TXA before 1 hour of surgery	Placebo	Intra-operative blood loss, postoperative edema, and ecchymosis severity scores	Marked decline in blood loss, edema, and ecchymosis in TXA as compared to controls
Merza (2021) [20]	2021	Bahrain	Double-blind RCT	54 (18 with one dose of dexamethasone, 18 patients with 3 doses of dexamethasone, 18 controls)	G1: 22.25 ± 4.12; G2: 23.06 ± 4.03; G3: 22.57 ± 3.86	8 mg/kg dexamethasone	No medication	Intra-operative blood loss, postoperative edema, and ecchymosis severity scores.	In comparison to control group 3, there was a significant reduction in periorbital edema and ecchymosis in groups l and 2. Group 2 had less periorbital edema and ecchymosis at the end of the first postoperative week than group 1
Gutierrez et al. (2014) [18]	2014	Turkey	A randomized, double-blinded, placebo-controlled trial	54 patients (28 one dose of dexamethasone, 26 controls)	28.5 ± 9.1	16 mg/kg	Saline	Intraoperative blood loss, post-operative edema, and ecchymosis severity scores	In between the experimental and control groups, there were no differences in ecchymosis, edema, and intraoperative blood loss

Quality Assessment

**Figure 2:**
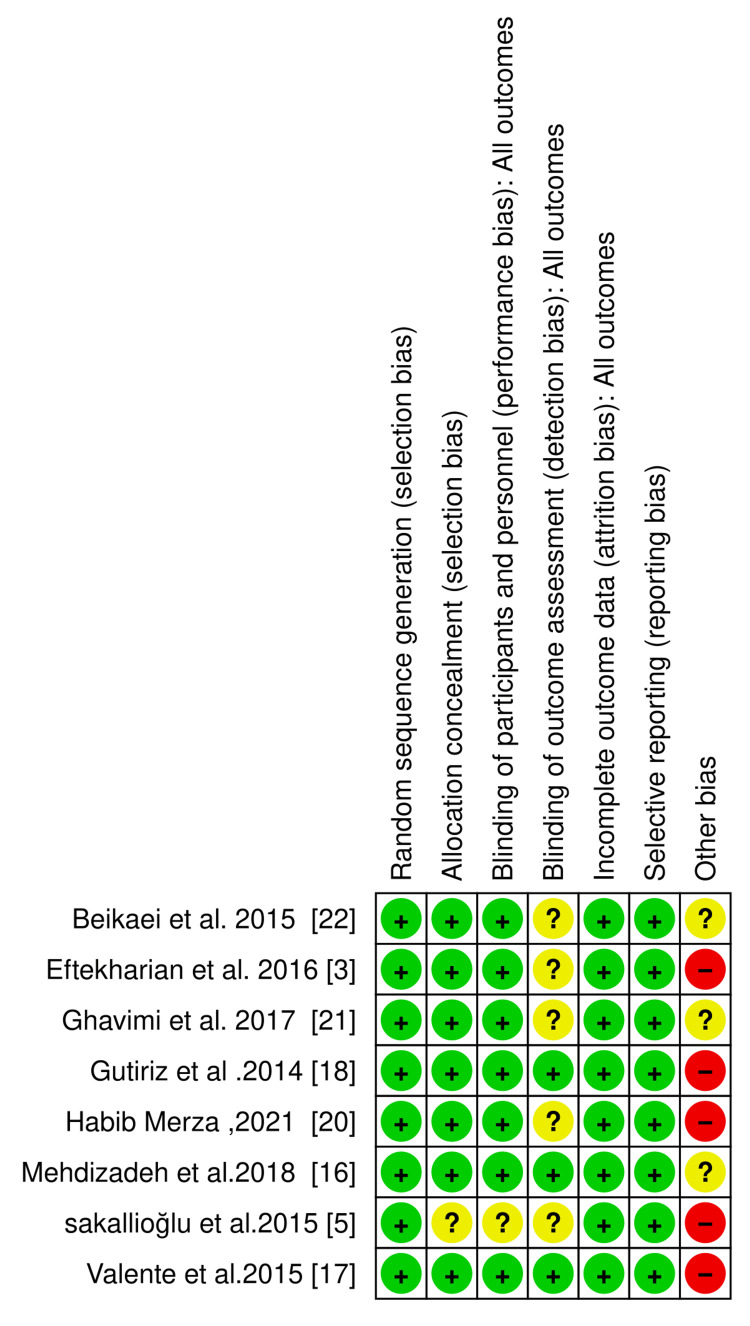
Risk of bias spotlight. References [3,5,16-18,20-22].

**Figure 3:**
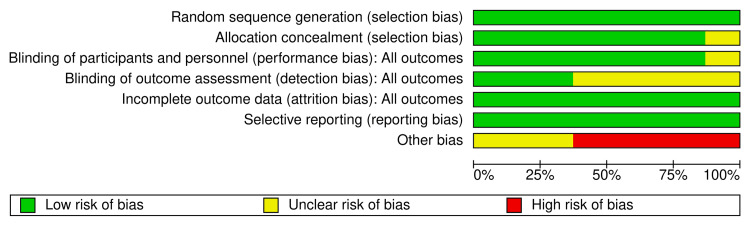
Risk of bias summary.

According to the quality assessment, one of the included studies [3] demonstrated an overall high ROB, primarily due to other methodological biases. Several studies, including Mehdizadeh et al. (2018) [16] and Ghavimi et al. (2017) [21], exhibited some concerns related to allocation concealment, blinding of personnel and participants, and outcome assessment. Most studies, however, displayed a low ROB in critical domains such as random sequence generation and handling of incomplete outcome data, including Beikaei et al. (2015) [22], Gutierrez et al. (2014) [18], Merza (2021) [20], and Sakallioğlu et al. (2015) [5]. The results are summarized in Figures 2, 3.

Efficacy Assessment of Tranexamic Acid and Dexamethasone

A meta-analysis was conducted to evaluate the effectiveness of tranexamic acid or dexamethasone in reducing intraoperative blood loss, edema, and ecchymosis compared to a placebo in patients undergoing rhinoplasty.

Bleeding Reduction

**Figure 4:**
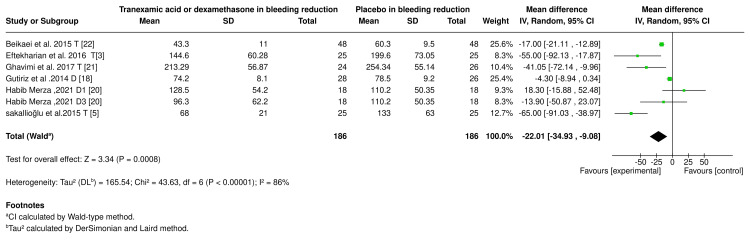
Blood reduction forest plot. D1 (one dose of dexamethasone), D3 (three doses of dexamethasone), T (tranexamic acid), and DT (dexamethasone and tranexamic acid). References [3,5,18,20-22].

The analysis included data from six studies, encompassing 186 participants in each group (experimental and placebo). The results revealed a high level of heterogeneity between trials (I² = 86%, P < 0.00001), suggesting variability in the study results. The meta-analysis revealed that tranexamic acid or dexamethasone significantly reduced bleeding by -22.01 ml (95% CI: -34.93 to -9.08) compared to placebo (P = 0.0008). Results are shown in the forest plot (Figure 4).

Edema Scores

**Figure 5:**
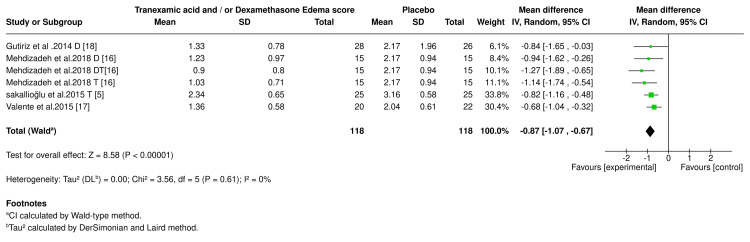
Edema score forest plot. D (dexamethasone), DT (dexamethasone and tranexamic acid), and T (tranexamic acid). References [5,16-18].

A meta-analysis was conducted to evaluate how effective tranexamic acid and/or dexamethasone were in reducing edema scores compared to placebo. The analysis included six studies with 118 participants in both the experimental and placebo groups. The results showed no heterogeneity between trials, indicating highly consistent findings across studies. The pooled effect indicated that tranexamic acid or dexamethasone significantly reduced the edema scores in the experimental group (-0.87, 95% CI: -1.07 to -0.67, P <0.001) compared to placebo. The edema score results of the experimental drugs are shown in the forest plot (Figure 5).

Ecchymosis Score

**Figure 6:**
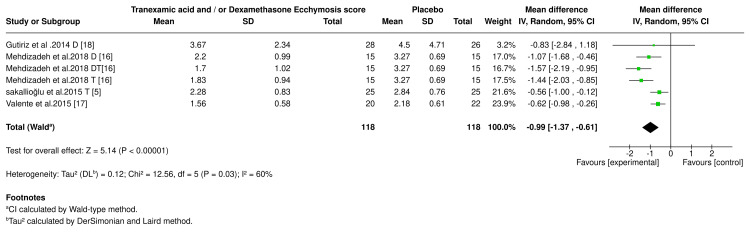
Ecchymosis score forest plot. D (dexamethasone), DT (dexamethasone and tranexamic acid), and T (tranexamic acid). References [5,16-18].

A meta-analysis was conducted to assess the effectiveness of tranexamic acid and/or dexamethasone in reducing ecchymosis scores compared to placebo. The analysis included six studies with 118 participants in both the experimental and placebo groups. The results revealed a moderate heterogeneity between trials (I² = 86%, P < 0.00001). This suggests some variability in the outcomes across studies. The pooled effect indicated that tranexamic acid or dexamethasone significantly reduced the ecchymosis score in the experimental group (-0.99, 95% CI: -1.37 to -0.61; P <0.001) compared to placebo. The ecchymosis score results of the experimental drugs are shown in the forest plot (Figure 6).

Discussion

The aim of the present systematic review and meta-analysis was to examine the efficacy and the role of tranexamic acid and dexamethasone independently or combined in lowering rhinoplasty patients' intraoperative blood loss, postoperative edema, and ecchymosis. Nine studies were included, most of which were of high quality and low ROB. The meta-analysis showed that tranexamic acid and dexamethasone can significantly reduce intraoperative blood loss, postoperative edema, and ecchymosis compared with control groups. With a significant effect size and high overall quality of evidence, rhinoplastic surgeons should consider using tranexamic acid and dexamethasone when performing rhinoplasties.

The role of tranexamic acid in decreasing surgical blood loss is well known, with its beneficial use being shown in cardiac surgery, obstetrics, and orthopedic surgery [2]. Corticosteroids may be administered at various doses during rhinoplasty. They can decrease swelling by reducing vascular permeability, diminishing inflammatory metabolites, and inhibiting lymphocyte and neutrophil migration. Dexamethasone sodium phosphate (10 mg) is one of the most potent anti-inflammatory corticosteroids; the onset is fast, and its effects are moderately long-lasting. It has a biological half-life of 72 hours [17].

A double-blind randomized placebo-controlled trial by Beikaei et al. (2015) [22] showed that one intravenous TXA bolus dose (10 mg/kg) upon induction of anesthesia significantly reduced intraoperative bleeding in patients undergoing open rhinoplasty. This finding was further confirmed by a study by Eftekharian et al. (2016) [3]. Preoperative oral administration of 1 g TXA significantly decreased blood loss in rhinoplasty patients. Both intravenous and oral tranexamic acid administration have demonstrated effectiveness, providing flexibility in administration routes. The consistent outcomes of these studies suggest that TXA is a valuable tool for hemostasis in rhinoplasty, potentially reducing the need for blood transfusions and improving surgical field visibility.

Although TXA has an evident role in decreasing intraoperative bleeding, there is less conclusive data on how TXA and dexamethasone affect ecchymosis and postoperative edema. Some studies indicate similar efficacy for both drugs in reducing these complications. For instance, Mehdizadeh et al. (2018) [16] conducted a prospective, triple-blinded randomized study and found no statistically significant difference in preventing or decreasing periorbital edema and ecchymosis between groups that received dexamethasone, TXA, or a combination of both, suggesting comparable effectiveness. This finding is supported by Sakallioglu et al. (2015) [5], who observed no significant difference in the reduction of periorbital edema and ecchymosis between patients who received oral TXA and those who received intravenous methylprednisolone. These findings suggest that both TXA and dexamethasone might be viable options for managing postoperative edema and ecchymosis, though further research is needed to confirm their comparative effectiveness and determine optimal dosage timelines.

The included studies show conflicting evidence regarding the effectiveness of dexamethasone alone in reducing ecchymosis and postoperative edema. Some studies, such as that by Valente et al. (2015) [17], support the use of dexamethasone, reporting a reduction in ecchymosis and edema at seven days after rhinoplasty with preoperative intravenous administration. Similarly, Merza (2021) [20] observed a significant reduction in ecchymosis and periorbital edema in groups that received dexamethasone in comparison to the control group. Studies such as Gutierrez et al. (2014), Mehdizadeh et al. (2018), and Valente et al. (2015) [16-18] consistently demonstrated reductions in ecchymosis compared to placebo. These results indicate that TXA and dexamethasone can effectively minimize postoperative bruising, potentially improving the aesthetic and recovery outcomes for patients undergoing rhinoplasty. These differences could be attributed to variations in study design, dosage regimens, and the timing of outcome assessments. Therefore, the effectiveness of dexamethasone in reducing postoperative edema and ecchymosis requires further clarification.

Despite the potential benefits of both TXA and dexamethasone individually, the included studies do not provide strong evidence for the superiority of combined therapy. A study by Mehdizadeh et al. (2018) [16] found no significant additional benefit from combining dexamethasone and TXA in reducing periorbital edema and ecchymosis. This finding suggests that combining these medications might not offer substantial advantages over using them individually. However, further research is needed to confirm this observation and explore potential benefits in other aspects of postoperative recovery.

Limitations

Despite the comprehensive assessment of the intervention in this review and meta-analysis, the study could be strengthened by incorporating more trials with larger and more diverse populations. Thus, more clinical trials should be conducted to validate these findings. It would be beneficial for future studies to incorporate standardized outcome measures and explore the long-term effects of these interventions on patient satisfaction and quality of life. Furthermore, investigating the cost-effectiveness of different treatment strategies could provide valuable insights for clinical decision-making.

## Conclusions

In conclusion, this systematic review and meta-analysis emphasized that tranexamic acid and dexamethasone administration before and during the rhinoplasty can remarkably reduce intraoperative bleeding, postoperative edema, and ecchymosis among patients undergoing rhinoplasty. With increasing efforts to conserve blood products, plastic surgeons should consider using tranexamic acid and dexamethasone in patients undergoing primary elective rhinoplasty. It could potentially lead to a better prognosis without burdening the patient with discomfort, pain, and high costs.
